# Taekwondo motion image recognition model based on hybrid neural network algorithm for wearable sensor of Internet of Things

**DOI:** 10.1038/s41598-023-40169-7

**Published:** 2023-08-11

**Authors:** Xiaotong Lu

**Affiliations:** Physical Education Institute, Yongin University, Yongin, 17092 South Korea

**Keywords:** Engineering, Materials science

## Abstract

Conventional IoT wearable sensor Taekwondo motion image recognition model mainly uses Anchor fixed proportion whole body target anchor frame to extract recognition features, which is vulnerable to dynamic noise, resulting in low displacement recognition rate of motion image. Therefore, a new IoT wearable sensor Taekwondo motion image recognition model needs to be designed based on hybrid neural network algorithm. That is, the wearable sensor Taekwondo motion image features are extracted, and the hybrid neural network algorithm is used to generate the optimization model of the wearable sensor Taekwondo motion image recognition of the Internet of Things, so as to achieve effective recognition of Taekwondo motion images. The experimental results show that the designed wearable sensor of the Internet of Things based on the hybrid neural network algorithm has a high recognition rate of the motion image displacement of the Taekwondo motion image recognition model, which proves that the designed Taekwondo motion image recognition model has good recognition effect, reliability, and certain application value, and has made certain contributions to optimizing the Taekwondo movement.

## Introduction

Taekwondo is an official Olympic event^1^, which evolved from Hualangdao in North Korea, and later developed into a martial art popular in Asia for a long time. In the process of Taekwondo, athletes often use both hands and feet to fight effectively. Early Taekwondo and coaches mainly judged athletes’ movements with the naked eye^2^, which was easily affected by subjective factors, leading to inaccurate final evaluation results. In the context of informatization, the evaluation of Taekwondo competitions has also been gradually upgraded, and advanced processing tools such as computers are used for evaluation^3^. However, due to the influence of complex action features of Taekwondo, its moving image recognition is difficult, and it needs to be completed through an effective moving image recognition model.

Moving image recognition is an advanced computer perception technology, which can combine the interaction state between human and computer to complete the recognition, thus generating an effective moving image recognition model^4^. To improve the recognition effect of moving images, it is necessary to capture human behavior perception data and set reasonable recognition parameters^5^. At present, many scholars at home and abroad are studying the problem of motion recognition perception, and put forward a variety of motion image perception recognition assumptions. However, due to the lack of relevant experience^6,7^, the recognition effect of most existing motion image recognition models is general.

Early in the process of human motion image recognition, a special camera was mainly used. This camera can take effective moving image sequences to identify human motion^8^. At this time, the camera is also called perception camera.With the progress of computer vision technology, in order to obtain the human motion image data^9,10^ from all angles, more and more perceptual cameras are used, and the total number of motion image sequences taken by cameras is increasing, so the recognition effect is relatively improved. However, research shows that the recognition limitations of the above recognition methods are large, and they are vulnerable to light, perception camera location, occlusion and other factors, resulting in high unique recognition deviation^11^. In addition, their recognition privacy is relatively intrusive, and they are not suitable for use in some scenes. In order to solve the above problems, this paper constructs a new wearable sensor Taekwondo motion image recognition model based on the hybrid neural network algorithm.

## Design of wearable sensor of Internet of Things based on hybrid neural network algorithm Taekwondo motion image recognition model

### Extraction of wearable sensor Taekwondo motion image features

Wearable sensors are sensor devices that can be worn on the body to collect data. Several types of wearable sensors that can be used for feature extraction include:

1. Accelerometers—Wearable accelerometers typically measure the acceleration and direction of body movement, which can be used to measure motion characteristics such as step count, walking speed, and activity intensity.
2. Gyroscopes—Gyroscopes can measure rotational movement of the body, such as bending, rolling, and spinning, and can be used to detect motion or posture changes.
3. Heart rate sensors—Heart rate sensors can measure changes in heart rate and the relationship between heart rate and other exercise or activity.
4. Temperature sensors—Temperature sensors can measure skin surface temperature changes, which can be used to detect changes in body temperature and other physiological features.
5. EMG sensors—Electromyography (EMG) sensors can measure the electrical signals generated when muscles contract, which can be used to detect muscle fatigue and activity level.
6. Body pressure sensors—Body pressure sensors can measure the pressure distribution of various parts of the body, which can be used to detect changes in body position and posture.
7. Optical sensors—Optical sensors can measure the reflection light intensity on the skin surface, which can be used to detect physiological features such as skin color, blood flow, and oxygenation.

The above sensors can be used to collect motion, physiological, and environmental data, and perform feature extraction. By processing and analyzing these data, various features such as human posture, movement behavior, and physiological conditions can be extracted and recognized. This article uses wearable sensors to extract Taekwondo motion image features.

Before moving image recognition, image pre-processing is required, which may be interfered by many factors during moving image transmission, resulting in recognition noise^12^. Therefore, image pre-processing is required before extracting moving image features to reduce noise. That is to use the average calculation method to divide a processing range^13^, screen effective change points, conduct noise removal processing, solve the problem of image blur, reduce image noise, and increase image clarity^14,15^. In addition, the computer can also be used to decompose the motion steps in the moving image, divide the motion details to achieve feature differentiation^16^, extract the comprehensive features of the image, and image feature differentiation $y_{ij}$ as shown in (1) below.

1 $$ y_{ij} = \sum\limits_{S = 1}^{S} {\sum\limits_{T = 1}^{T} {W_{st} x_{i - s + 1} } } . $$

In formula (1), $W_{st}$, $x_{i - s + 1}$ represents the coordinate element of the moving image, S and T represent the size of the recognition filter respectively. After feature extraction of moving image, because the dimension of feature image cannot be determined, it is necessary to select improved neural network parameters, carry out dimension reduction processing, connect sampling layers^17^, reduce the complexity of dimension reduction calculation, shorten the dimension reduction range, and improve the calculation accuracy. At this time, we can use mathematical methods to describe the Taekwondo moving image^18^, and use formula (2) to calculate the corresponding gray value of pixels $V_{gray}$

2 $$ V_{gray} = \frac{{sa \times V_{red} + sb \times V_{green} + sc \times V_{blue} }}{{sa{ + }sb + sc}}. $$

In formula (2), $V_{red}$, $V_{green}$, $V_{blue}$ they represent different color brightness values of moving images, $sa,sb,sc$ they represent the total content of pixels in the moving image^19^. At this time, the gray value of pixels in the moving image recognition can be used $f(x_{m} ,y_{m} )$ in order to improve the effectiveness of extracted features^20^, it is necessary to calculate the original weighted average details of the moving image $g(x,y)$, as shown in (3) below.

3 $$ g(x,y) = \frac{1}{M}\sum\limits_{(i,j) \notin S} {w(i,j)f(x{\text{ + i}},y + j)} . $$

In formula (3), $w(i,j)$ represents the filtered moving image, M represents the number of pixels in the neighborhood, and i and j represent the change threshold^21^ of the moving image. After the above steps are completed, the comprehensive features of the image can be extracted $f(X)$, as shown in (4) below.

4 $$ f(X) = {\text{sgn}} \left( {\sum\limits_{i = 1}^{N} {\alpha g(x)} } \right). $$

In formula (4), $\alpha$ represents the iteration threshold set by the identification, $g(x)$ represents the feature recognition index of the moving image. Using the above steps can improve the recognition accuracy of the moving image and reduce the impact of external interference on the final recognition result.

### Generate the optimal model of Taekwondo motion image recognition for wearable sensors of the Internet of Things based on hybrid neural network algorithm

**Figure 1:**
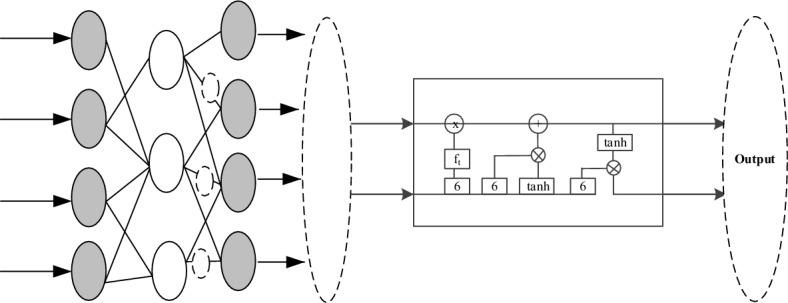
BP and LSTM hybrid neural network structure.

In order to solve the problem that the anchor frame of anchor fixed proportion whole body target is affected by dynamic noise when extracting recognition features, which leads to the low recognition rate of motion image displacement^22,23^, this paper generates an optimization model for Taekwondo motion image recognition of Internet of Things wearable sensor based on hybrid neural network algorithm. The method designed in this paper selects BP and LSTM hybrid neural networks for motion image recognition and judgment^24^, as shown in Fig. 1.

At this time, the standard mean value of each joint point measured by IMU needs to be calculated $\sigma$, as shown in (5) below.

5 $$ \sigma { = }\frac{{\sum\nolimits_{b = 1}^{18} {\sqrt {\frac{{\sum\nolimits_{b = 1}^{n} {(a_{1} - a)^{2} } }}{n}} } }}{18}. $$

**Figure 2:**
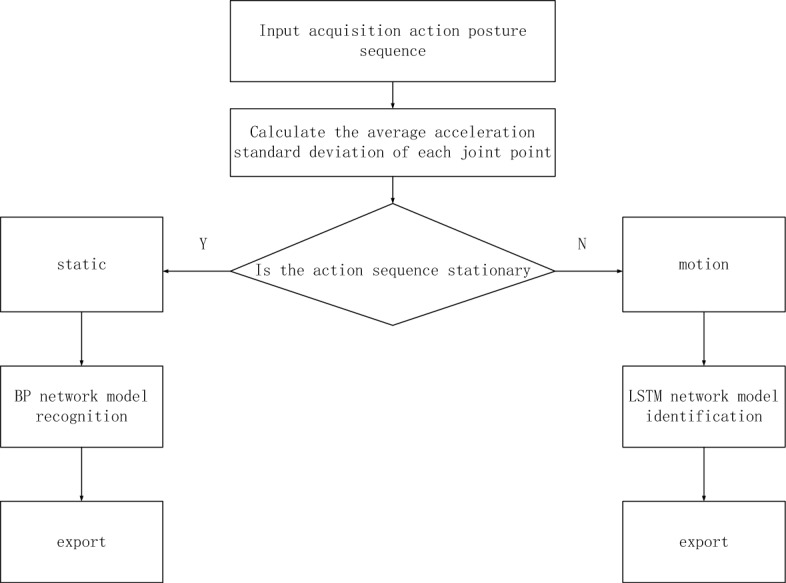
Moving image recognition process.

In formula (5), $b$ represents the number of joint points, $a_{1}$ represents the acceleration value of the joint point, $a$ represents the average acceleration value, $n$ representing the number of sequence frames, combined with the above standard mean^25^, we can judge the motion stillness category of the moving image. At this time, the generated moving image recognition process is shown in Fig. 2.

It can be seen from Fig. 2 that training samples can be input in combination with the above moving image recognition process, and the input value of hidden layer neurons^26^ can be calculated. At this time, the optimal model for Taekwondo moving image recognition of wearable sensors of the Internet of Things is built based on the hybrid neural network algorithm $E(w)$ as shown in (6) below^27^.

6 $$ E(w) = \frac{1}{2}\sum\nolimits_{n = s}^{L} {(y_{n} - y)^{2} } . $$

In formula (6), $y_{n}$ represents the hidden neuron connection weight, $y$ represents the input neuron connection weight^28^. Using the above built IoT wearable sensor Taekwondo motion image recognition optimization model can effectively obtain the motion recognition weight, output effective image sequence recognition results^29,30^, and improve the reliability of motion image recognition.

## Experiment

In order to verify the recognition effect of the designed wearable sensor Taekwondo motion image recognition model based on the hybrid neural network algorithm, this paper built an experimental platform, and compared it with the conventional wearable sensor Taekwondo motion image recognition model, and carried out experiments, as follows.

### Experiment preparation

**Figure 3:**
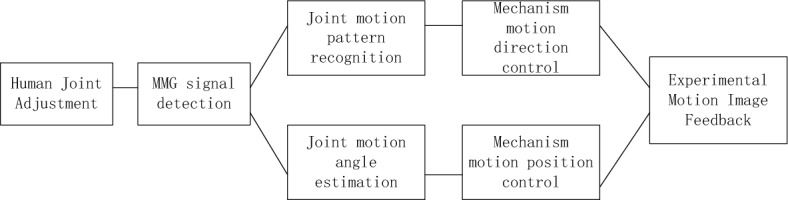
Basic flow of experiment.

Combined with the experimental requirements, this paper selects the Solid Works 3D virtual simulation platform as the experimental platform. The experimental platform is equipped with mainstream CAD analysis software, with good comprehensive performance. In the process of Taekwondo movement, the movement modes and basic movement angles of human joints are different for different movements. Therefore, the movement trend of human joints can be predicted according to the movement transformation trend of human joints to achieve coordinated motion control. Therefore, this experiment conducted motion estimation in combination with the MMG signal of human motion images, and generated the basic experimental process, as shown in Fig. 3.

**Figure 4:**
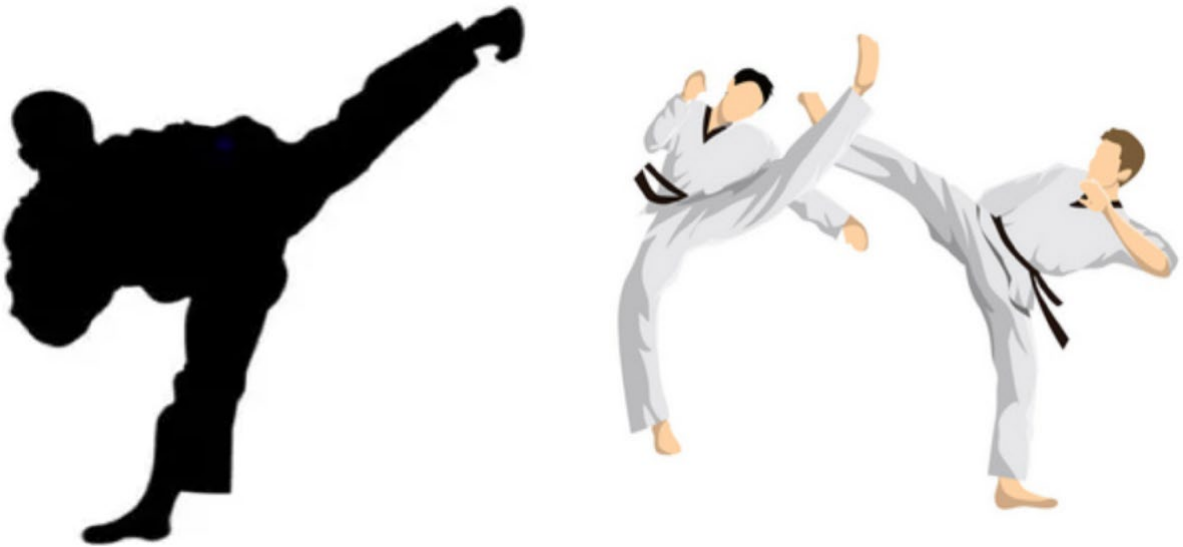
Taekwondo motion images.

It can be seen from Fig. 3 that during the experiment, the MMG signal of the image can be continuously detected, and the action simulation can be carried out. With the relevant motion angles as a reference, effective control algorithms can be used to correct, so as to collect Taekwondo motion images that meet the experimental requirements, as shown in Fig. 4.

At this time, the joint angle discretization parameters under different motion modes are shown in Table 1.

**Table 1:** Joint angle discretization parameters under different motion modes.

Sport mode	Motion state	Ankle joint angle discretization parameter	Knee joint angle discretization parameter	Hip joint angle discretization parameter
AS1-KL5	Alternate front and rear legs for quick attack	0.652	0.751	1.441
AS2-KU4	Slow kick attack with front and rear feet	0.781	0.623	1.323
AS3-HK3	Continuous step attack	0.551	0.568	1.285
AS4-KK9	Quick back defense	0.623	0.754	1.563
AS5-KL5	Quickly twist the waist and crotch with the right foot as the pivot	0.482	0.529	2.555
AS6-RL4	Using the sole of the foot as the focus, aggressive forward kicking	0.639	0.535	3.947
AS7-HK2	Heel rotates 120° with body	0.557	0.648	3.585
AS8-HQ8	Kick down and straighten your knees over your waist	0.533	0.588	2.426
AS9-QQ3	Hip extension and kick	0.681	0.465	5.339
AS10-HH9	Hip retraction and waist arch	0.528	0.314	3.544
AS11-QH4	Left foot support split leg	0.484	0.223	6.185
AS12-KX2	Turn around and swing your legs	0.554	0.144	3.229

It can be seen from Table 1 that moving image recognition data can be selected in combination with the above joint discretization parameters. In order to ensure the reliability of recognition, image data needs to be preprocessed, that is, select the PC association platform, adjust the main frequency of moving image processing to 2.5 GHz, and then the corresponding internal memory is 5 GB. Input the above obtained joint angle discretization parameters, calculate the discrete state frequency of different signal segments. After the above steps are completed, the joint angle basic signal graph can be generated to classify the experimental data. At this time, the segment slope and the number of association rules of each signal segment are shown in Table 2.

**Table 2:** Segment slope and number of association rules of experimental data.

Signal fragment	Mode status	Segment slope	Number of association rules	Image training coefficient	Support (%)	Confidence (%)
L1	I1-I2-C0	− 0.621	1453	0.15	1.27	84.54
L2	I2-I3-C1	− 0.602	1239	0.15	1.25	85.12
L3	I3-I4-C2	− 0.543	1358	0.25	1.51	81.84
L4	I4-I5-C3	0.365	1251	0.10	1.08	82.23
L5	I5-I6-C4	− 0.541	1232	0.12	1.51	83.55
L6	I6-I7-C5	0.412	1363	0.15	1.63	86.98
L7	I7-I8-C6	− 0.683	1251	0.25	1.58	75.28
L8	I8-I9-C7	0.746	1228	0.35	1.61	84.65
L9	I9-I10-C8	− 0.635	1344	0.40	1.36	72.84
L10	I10-I11-C9	− 0.667	1238	0.30	1.04	88.45
L11	I11-I12-C10	0.588	1384	0.35	1.58	83.39
L12	I12-I13-C11	0.553	1232	0.25	1.32	79.84

**Figure 5:**
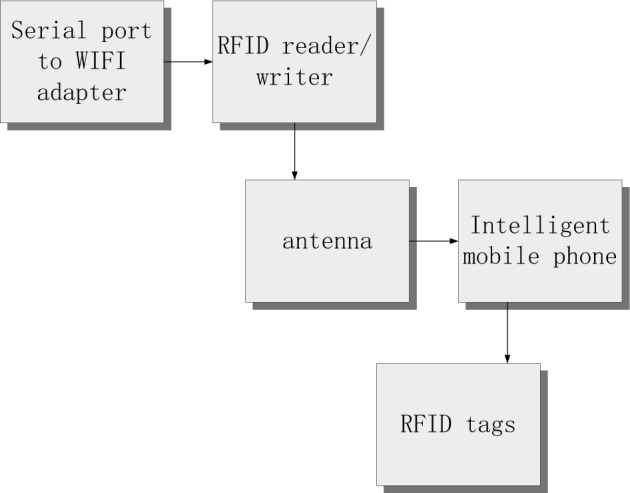
Connection diagram of experimental hardware device.

It can be seen from Table 1 that after the above experimental parameters are set, the experimental hardware can be connected. This paper selects the RFID Internet of Things wearable sensor as the experimental sensor. At this time, the schematic diagram of the experimental hardware device connection is shown in Fig. 5.

It can be seen from Fig. 5 that the core of the above experimental hardware is Impinj R2000 RFID reader writer, which is configured with a power supply of 9000 mA and several identification antennas. The transmission power of the identification antenna is 0–30 dbm, and the accuracy can be adjusted. During the experiment, it is necessary to ensure that the reading and writing frequency band of the core reader writer is within the specified range. In order to improve the effectiveness of recognition results, this experiment selects US FCC 47 CFG as the support, and sets the ETSI EN 302,208 image recognition standard. During the experiment, it is necessary to always ensure that the reader is in the Inventory working mode, and try to improve the read–write range of the reader. After the above hardware devices are connected, the experimental indicators can be selected and the recognition rate formula after moving image displacement can be designed $D$, as shown in (7) below.

7 $$ D = \frac{{W - W_{0} }}{R}. $$

In formula (7), $W$ represents the position of the base image after displacement, $W_{0}$ represents the position of the identified image before displacement, $R$ image preset displacement, the higher the recognition rate after moving image displacement, the better the recognition effect of the moving image recognition model. On the contrary, the lower the recognition rate after moving image displacement, the poorer the recognition effect of the moving image recognition model. Before the experiment process, it is also necessary to connect the MEMS inertial sensor to ensure that it meets the requirements of actual moving image recognition. The specifications and parameters of the sensor are shown in Table 3.

**Table 3:** Specifications and parameters of MEMS inertial sensor.

Parameter	Accelerometer	Gyroscope	Magnetometer
Coordinate axis	3-Axis	3-Axis	3-Axis
range	± 16 g	0.001035	± 4800 μT
ADC bits	16	16	14
Output quantity (Hz)	4000	8000	10
Nonlinearity (% of FS)	± 0.5	± 0.1	–
Noise density	100 μg\HZ	0.04°/HZ	–
Temperature sensitivity (%/°C)	± 0.026	± 4	–

Table 3 shows that the parameters of the above inertial sensors meet the experimental requirements. In addition to the above preparations, it is also necessary to set the relevant parameters of the hybrid neural network and prepare the experimental data set.

The hybrid neural network is a model that combines convolutional neural networks and fully connected neural networks. In the process of image recognition, the following parameters are usually set for the hybrid neural network:

1. *Input layer size* this refers to the size of the input image, which usually has three dimensions of length, width, and channel number, such as 224 × 224 × 3.
2. *Convolutional layer parameters* these include specifying the size of the convolution kernel, the number of convolution kernels, the step size, and the padding mode. These parameters determine the size of the output of the convolutional layer and the number of feature maps.
3. *Pooling layer parameters* these include specifying the size of the pooling kernel and the step size. These parameters determine the size of the output of the pooling layer and the number of feature maps.
4. *Fully connected layer parameters* these include specifying the number of neurons in the fully connected layer and activation function.

In the recognition process of the hybrid neural network, the first step is to input an image, which then undergoes a series of processing by convolutional layers and pooling layers. The feature maps are continuously reduced in size while extracting different features of the image. Multiple feature maps are then merged into a single vector, and classification is performed through the fully connected layer, ultimately resulting in the classification result of the image.

The Taekwondo motion image recognition data set used in this article is as follows:

1. *KTH-TIPS2-B* This data set contains 384 images of 6 different Taekwondo movements (front kick, back kick, turn kick, side kick, high side kick and low side kick). Each movement was performed by two different actors, and each actor performed it 16 times.
2. *KyungHee TaeKwonDo Dataset* This data set contains 1010 images of 10 different Taekwondo movements (push hands, wrist palm strike, front kick, knee kick, backward kick, back kick, side kick, single leg jump kick, consecutive kick and bottom kick). Each movement was performed by different actors.
3. *NTU RGB* + *D Dataset* This data set contains 56 different actions, including 11 different Taekwondo movements. Each movement was performed by 40 different actors, and was captured under RGB and depth sensors.

After all experimental preparation devices have been connected, subsequent motion image recognition experiments can be conducted.

### Experimental results and discussion

In combination with the above experimental preparations, we can carry out subsequent experiments on the wearable sensor of the Internet of Things for Taekwondo motion image recognition. That is, in the built experimental platform, the wearable sensor Taekwondo motion image recognition model based on the hybrid neural network algorithm designed in this paper and the conventional wearable sensor Taekwondo motion image recognition model are respectively used for motion image recognition, and the public formula (1) is used to record the motion image displacement recognition rate of the two methods in different motion modes. The experimental results are shown in Table 4.

**Table 4:** Experimental results.

Sport mode	The motion image displacement recognition rate of the Taekwondo motion image recognition model based on hybrid neural network algorithm for wearable sensors in the Internet of Things designed in this paper (%)	Motion image displacement recognition rate of conventional wearable sensor Taekwondo motion image recognition model (%)
AS1-KL5	94.84	71.51
AS2-KU4	95.39	75.36
AS3-HK3	93.64	72.94
AS4-KK9	95.43	63.23
AS5-KL5	98.88	75.52
AS6-RL4	98.66	64.84
AS7-HK2	96.28	71.36
AS8-HQ8	92.84	52.95
AS9-QQ3	94.56	73.14
AS10-HH9	95.85	65.25
AS11-QH4	98.14	64.98
AS12-KX2	95.51	68.38

Table 4 shows that the displacement recognition rate of the motion image of the wearable sensor Taekwondo motion image recognition model designed in this paper based on the hybrid neural network algorithm is high in different motion modes, while the displacement recognition rate of the motion image of the conventional wearable sensor Taekwondo motion image recognition model is relatively low. It proves that the wearable sensor Taekwondo motion image recognition model designed in this paper has good recognition performance, effectiveness and certain application value.

By comparing the contrast, clarity, and recognition time of the denoised images using other algorithms, the advantages of the proposed algorithm in identifying image features were verified.

1. *Contrast* refers to the measurement of the different brightness levels between the brightest white and darkest black areas in an image. The greater the difference range, the greater the contrast, and vice versa. This value has no standard definition, therefore, in this study, it is set to 35 based on human comfort level.
2. *Clarity* is the average gradient of the image, which can sensitively reflect the ability of the image to express small contrasts. Information entropy represents the size of the information contained in the image. With the increase of the information entropy value, the information value contained in the feature image also increases, resulting in higher clarity.
3. *Recognition time* reflects the recognition efficiency of each model, and this value is recorded by the computer.

The enhancement effect comparison results between the method proposed in this paper and traditional methods are shown in Table 5.

**Table 5:** Experimental results.

Comparison item	Precision/%	Contrast ratio	Information entropy	Time taken/s
Initial image	–	25.76	6.74	8.05
The motion image displacement recognition rate of the Taekwondo motion image recognition model based on hybrid neural network algorithm for wearable sensors in the Internet of Things designed in this paper (%)	95.39	28.38	9.87	6.47
Motion image displacement recognition rate of conventional wearable sensor Taekwondo motion image recognition model (%)	85.49	37.46	8.21	10.87

From Table 5, it can be concluded that the larger the average value during image recognition, the better the ability to improve image brightness, and the accuracy of the proposed method is 95.39%, the highest among compared algorithms. This indicates that the proposed algorithm has a better ability to enhance image brightness, ensuring higher contrast and information entropy values. This shows that while the image recognition effect is good, it also contains more information. In terms of algorithm running time, the proposed algorithm also has the shortest time. The experimental results above prove that the proposed algorithm has obvious advantages in identifying image features.

## Conclusion

Taekwondo is a common sport, which has many trainers in various countries. The motion pattern of Taekwondo is complex and difficult to identify effectively, so it needs to be comprehensively evaluated through the sensor photography. In recent years, computer vision technology has developed more and more rapidly in China, and some researchers have applied it to the recognition of Taekwondo motion images. The conventional recognition model of Taekwondo motion image has poor recognition effect and does not meet the current recognition requirements. Therefore, this paper designs a new wearable sensor Taekwondo motion image recognition model based on the hybrid neural network algorithm. The experimental results show that the wearable sensor Taekwondo motion image recognition model has good recognition performance, reliability and certain application value, and has made certain contributions to optimizing Taekwondo sports skills.

At present, hybrid neural network algorithms have achieved certain results, but there is still room for improvement. Future research will use many state-of-the-art deep learning models such as Alexnet, Googlenet, and Xception to classify images and improve algorithm performance.

## Data Availability

The datasets used and/or analysed during the current study are available from the corresponding author on reasonable request.
